# Modeling learning engagement in AI-supported education: a crossover enabler–inhibitor framework

**DOI:** 10.3389/fpsyg.2026.1859401

**Published:** 2026-06-10

**Authors:** Saleh Alwahaishi, Waqas Ahmed

**Affiliations:** 1 Department of Information Systems and Operations Management, King Fahd University of Petroleum and Minerals, Dhahran, Saudi Arabia; 2 IRC for Finance and Digital Economy, KFUPM Business School, King Fahd University of Petroleum & Minerals, Dhahran, Saudi Arabia

**Keywords:** AI learning engagement, cognitive fatigue, feedback overload, learning motivation, technology self-efficacy

## Abstract

Artificial intelligence is reshaping how learners interact with content, feedback, and motivation. Yet the psychological processes that support or disrupt engagement in AI-powered environments remain underexplored. This study develops a crossover model to explain how enabling beliefs and emotional strain jointly influence learning engagement. Drawing from self-determination, cognitive load, and social cognitive theories, the model tests how performance expectancy and technology self-efficacy function alongside feedback overload and AI learning anxiety. Learning motivation and AI fatigue are positioned as mediators linking these factors to behavioral engagement. Data were collected from 251 learners with experience using AI-driven educational tools. Structural equation modeling revealed that learning motivation is the most powerful predictor of engagement. Performance expectancy increases motivation, while feedback overload weakens it. Technology self-efficacy has no significant effect on either motivation or fatigue. Fatigue, although linked to anxiety and overload, did not directly reduce engagement. These results suggest that motivation, not depletion alone, drives sustained learning behavior. This study contributes by reframing inhibitors as threats to motivation rather than isolated stressors. It offers practical insights for designing AI systems that preserve psychological energy, minimize cognitive strain, and reinforce learning purpose. Engagement is not automatic. It must be carefully engineered within the emotional architecture of intelligent systems. For AI-system designers, this means embedding motivation-aware dashboards, adaptive feedback controls, and anxiety monitoring features to protect and sustain learner engagement.

## 1 Introduction

The growing presence of artificial intelligence (AI) in education has outpaced our understanding of its effects on student learning and engagement. Learning management systems, recommendation engines, and generative AI tools have transformed content delivery into a dialogic, feedback-rich process (Chang and Sun, 2024; Qazi et al., 2024). At the core of this transformation is the assumption that students will meaningfully engage with these systems. Such systems assume that learners will perceive value, sustain motivation, and meaningfully integrate AI-generated feedback into their learning behavior (Chen et al., 2026; Ji and Ye, 2026; Li et al., 2026). Yet, as technology evolves, our understanding of what truly enables or inhibits AI-mediated engagement has not kept pace (Chen and Lin, 2026). Most contemporary research treats learner engagement as a direct outcome of enabling conditions: technological self-efficacy, perceived usefulness, and motivational readiness (Liwanag and Galicia, 2023; Ma et al., 2026; Wang et al., 2022). This view draws heavily from established technology acceptance and motivation frameworks. However, growing evidence from AI-enhanced settings suggests that engagement is not merely enabled. It is also systematically undermined by cognitive and emotional overload (Hina et al., 2025; Jiang et al., 2026; Liu et al., 2025). Feedback intended to support learning may overwhelm; autonomy meant to empower may paralyze (Wang, 2026). Despite their centrality to AI interaction, these inhibitory forces have received limited theoretical attention in educational models.

The enabler–inhibitor framework emerged in broader information systems research to address this imbalance (Cenfetelli, 2004; Liang et al., 2025). It recognizes that user behavior is shaped by both reinforcing and restricting forces. In educational contexts, this lens has been only modestly applied (Singh et al., 2025). Recent studies have begun identifying enablers such as performance expectancy and self-efficacy, as well as inhibitors like AI learning anxiety (Balakrishnan et al., 2024; Wang et al., 2022). However, most research treats these factors as parallel and independent, assuming that enablers promote engagement and inhibitors impede it, with minimal interaction between the two. This static binary framing is poorly suited for AI-enhanced learning environments, which are psychologically complex and behaviorally demanding. Learners rarely experience technological affordances in isolation. Motivation, cognitive load, self-efficacy, and system feedback often converge, triggering intertwined emotional and behavioral responses (Alexander et al., 2026). A student facing performance pressure and system overload, for instance, may not just experience fatigue. They may begin to question their ability to engage, even if they initially believed in the value of the system. Conversely, learners with strong self-efficacy may not only persist more but also resist the demotivating effects of technical strain. These nuanced psychological interactions remain largely under-theorized in current engagement models.

This study addresses that gap by advancing a crossover enabler–inhibitor model. Unlike dual-path frameworks that conceptualize enablers and inhibitors as independent inputs, the crossover model explores how inhibitors such as feedback overload and anxiety do more than create fatigue. They suppress the very motivational mechanisms that drive engagement (Li et al., 2026). Drawing from cognitive load theory (Paas et al., 2003; Sweller, 1988), we propose that overload reduces perceived controllability and autonomy, which are central to motivation in self-directed AI learning. In contrast, enablers like technology self-efficacy may buffer emotional fatigue, offering resilience in high-autonomy learning settings (Liwanag and Galicia, 2023; Schunk and DiBenedetto, 2020). This shift is more than technical. It repositions motivation as a psychological fault line, both the driver of learning behavior and the point of greatest vulnerability. Although engagement is often seen as the endpoint of motivation, we suggest that in AI-mediated learning, motivation itself is under threat. Prior work in AI anxiety, organizational overload, and educational fatigue supports this interpretation, yet such dynamics have rarely been modeled explicitly (Chen et al., 2024; Hina et al., 2025). Most existing frameworks fall short of explaining how enablers and inhibitors might actively interfere with one another, creating unpredictable effects on student behavior.

Recent educational psychology research increasingly conceptualizes learning engagement as a self-regulated psychological process shaped not only by technology perceptions, but also by learners’ cognitive and emotional regulation capacities. Studies in technology-supported learning environments further suggest that academic engagement is closely associated with learners’ higher-order thinking processes and emotion regulation strategies. In AI-supported learning environments, learners continuously manage attention, uncertainty, motivational fluctuations, and cognitive demands while interacting with adaptive systems. Engagement therefore emerges not solely from technological affordances, but from the learner’s ability to regulate psychological resources under varying instructional and emotional conditions. This broader perspective aligns with emerging work connecting self-determination theory, emotion regulation, and technology-supported learning engagement (Ryan and Deci, 2000; Stalmach et al., 2025). Recent AI education research has also begun integrating self-determination theory with technology acceptance perspectives to explain how perceived usefulness, autonomy, and intrinsic motivation jointly shape engagement with intelligent learning systems.

To explore this, we integrate self-determination theory (Deci and Ryan, 2012), cognitive load theory, and social cognitive perspectives on efficacy and emotional regulation. These frameworks, when combined, offer a foundation to study how beliefs, feedback, and system strain co-produce engagement outcomes (Guan et al., 2024; Ji and Ye, 2026). Yet their synthesis in AI learning contexts remains limited, especially in explaining how engagement is sustained or disrupted under competing psychological pressures. A further limitation of existing studies is their outcome focus. Most research emphasizes intention to use, satisfaction, or system continuance, overlooking the more behaviorally grounded construct of learning engagement (Singh et al., 2025). By focusing on AI learning engagement behavior (LEB), the cognitive, emotional, and behavioral investment in learning tasks mediated by AI, this study addresses a major empirical gap. Few studies have applied the enabler–inhibitor lens to LEB specifically, even though it is arguably the most meaningful indicator of effective system interaction (Xu et al., 2024; Zhang and Li, 2024). Understanding how enablers and inhibitors interact to shape actual engagement behavior, not just intention, is critical for guiding both theory and practice in AI-supported education.

We propose that feedback overload does not merely exhaust learners, it disrupts motivation. Similarly, anxiety toward AI tools reduces self-regulatory confidence, suppressing both cognitive and emotional readiness. On the other hand, enablers like performance expectancy and self-efficacy may enhance motivation and insulate learners from psychological strain. This integrated model positions motivation as a dynamic mediator, shaped, supported, and destabilized by a constellation of internal and external forces. This study contributes a refined theoretical framework in which engagement is not the mere result of motivation, but a function of how motivation survives under cognitive pressure. It reconceptualizes inhibitors not only as direct threats to learning but as disruptors of the very enablers meant to sustain it. And it offers a behavioral outcome, AI learning engagement, that more accurately reflects what learners do, not just what they intend. In reframing motivation as both a catalyst and casualty of AI-mediated learning, this study helps recenter engagement theory around the lived psychological complexity of intelligent educational environments. Accordingly, the present study seeks to address the following research questions:

*RQ1*: What is the relationship between enabling and inhibiting factors in shaping AI learning engagement behavior?

*RQ2*: Does a crossover between enabler and inhibitor aspects influence learner motivation and engagement outcomes?

## 2 Literature review

### 2.1 Enabler- inhibitor Lens

The enabler–inhibitor framework is pivotal to this research because it strikes a balance for the analysis of both factors that influence student participation with regard to AI-augmented learning. This differs from other models which focus solely on adoption or utility because this model accounts for the cognitive, emotional, and behavioral conflicts within a learner. As pointed out by Balakrishnan et al. (2024), enablers like ease of use and performance benefits also come with inhibitors such as risk perception and resistance to change. In educational settings, both sides of the equation need to be addressed, particularly when learners psychologically tire and feedback loops silently erode persistence (Farooq et al., 2024; Huang et al., 2023). In AI-mediated learning, this encapsulates the dualistic interplay that shapes learner engagement; the facilitators that aid in adoption and deepening engagement, and barriers that impede persistence. Understanding these counterbalancing forces is crucial for designing effective adaptive learning ecosystems that are optimally efficient and distinctly human-centered (Hina et al., 2025; Liu et al., 2025; Tandon et al., 2024).

Within the present framework, each theory contributes a distinct explanatory role. Self-Determination Theory explains how autonomy, competence, and motivational activation shape sustained engagement in AI-supported learning environments (Ryan and Deci, 2000; Deci and Ryan, 2012). Social Cognitive Theory explains how efficacy beliefs influence learners’ confidence in navigating technology-mediated tasks (Bandura, 1986). Cognitive Load Theory clarifies how excessive informational demands and feedback saturation deplete cognitive resources and generate fatigue (Sweller, 1988; Paas et al., 2003). Finally, the Unified Theory of Acceptance and Use of Technology explains how performance expectancy shapes learners’ perceptions of the educational value of AI systems (Venkatesh and Davis, 2003). Together, these perspectives provide a psychologically integrated explanation of how enabling beliefs and inhibitory pressures jointly shape engagement behavior in AI-mediated learning contexts.

The Dual-Factor Theory by Herzberg and Bernard Mausner (1959) focused initially on motivators and hygiene factors regarding job satisfaction, but has been modified in behaviorial fields, such as education (Suldo et al., 2016) and consumer behavior (Park et al., 2018). Within Information Systems, this theory has been used to differentiate between enablers that drive technology adoption and inhibitors that hinder its progress. Cenfetelli (2004) furthered this due by proposing a matrix categorizing outcomes of IS usage based on enabling or inhibiting perceptions. In the current research, Dual-Factor Theory is used to frame the enabler–inhibitor framework of learning engagement behavior within AI-enhanced educational contexts. Technology Self-Efficacy, Performance Expectancy, and Learning Motivation act as sustained impact drivers of adopting AI-enabled learning. On the other hand, AI Learning Anxiety, Feedback Overload, and AI Fatigue serve as cognitive and emotional ‘frictions’ that deter engagement.

Bandura (1986) defined Social Cognitive Theory (SCT) by self-efficacy, observational learning, and outcome expectations as determinants of behavior. In education, Technology Self-Efficacy determines how students’ self-efficacy impacts the use of AI tools for learning (Schunk and DiBenedetto, 2020). Alongside SCT, the Unified Theory of Acceptance and Use of Technology (UTAUT) outlines predictors of technology acceptance, such as Performance Expectancy and Effort Expectancy. Learners are likely to adopt and engage with technologies proactively if they perceive tangible benefits to their academic performance. UTAUT identifies Effort Expectancy, which posits that learners are encouraged to adopt and sustain use of AI-powered instructional materials that are perceived as easier to use. In this case, the combination of SCT and UTAUT offers a rich description about the enabler constructs and clarifies the interaction of cognitive beliefs and technology perceptions.

Cognitive Load Theory (CLT) suggests that learners’ cognitive resources within working memory are limited, and when the load reaches a certain threshold, engagement declines (Paas et al., 2003; Sweller, 1988). AI Learning Anxiety increases intrinsic load by elevating the burden of learning newer technological competencies. Feedback Overload aligns with extraneous load in that it offers perplexing information far beyond learners’ capacity to process. AI Fatigue stems from cognitive overload sustained over time. Hence, CLT offers a structured approach to understanding the interplay between specific cognitive obstacles posed by AI tools and student disengagement.

### 2.2 Variable and hypotheses

#### 2.2.1 Technology self-efficacy (TSE)

Technology self-efficacy (TSE) serves as a pivotal psychological driver concerning how users engage with digital learning systems. TSE is defined as a technology-enabled learner’s belief regarding their capability to perform the tasks and activities associated with the technology. TSE fosters autonomous learning that feeds intrinsic and extrinsic motivation. Bandura’s social cognitive theory posits motivation is influenced by self-efficacy, so Compeau and Higgins (1995) claim self-efficacy strategies impact an individual’s selection, effort, and perseverance during cognitively demanding activities. Bergdahl and Sjöberg (2025) illustrated that higher levels of TSE are associated with greater adoption of AI tools and more proactive engagement. Zhang and Li (2024) similarly confirmed self-efficacy predicted motivation to participate in learning environments. The integration of TSE into learning motivation is further substantiated by studies on SRL strategies (Liwanag and Galicia, 2023; Nie et al., 2024; Xu et al., 2024). TSE has also been found to promote cognitive engagement through motivational channels (Li et al., 2024). In parallel, greater self-efficacy may buffer emotional exhaustion. Learners with higher TSE often report lower stress levels when engaging with complex systems, suggesting a negative relationship with AI fatigue. This is supported by Chang and Sun (2024), who emphasized that learners with stronger technology confidence demonstrated perseverance with minimal fatigue, and by Huang et al. (2023), who linked self-efficacy with reduced emotional exhaustion in digital learning environments. As such, TSE is positioned as both a motivational enabler and emotional regulator in AI learning, we hypothesize that:

*H1*: TSE positively influences LM

*H2*: TSE negatively influences AF

#### 2.2.2 Performance expectancy (PE)

Performance expectancy (PE) concerns the extent to which a user perceives a specific system or technology as likely to yield measurable benefits to his or her academic performance. In the context of learning environments imbued with artificial intelligence, PE operates as an important belief directly energizing students’ motivation. The expectation that technology will enhance understanding or outcomes motivates learners to engage with that technology even more. As detailed in Venkatesh and Davis (2003), performance expectancy is a distinct and key determinant of user motivation, behavioral intention, and adherence to newly introduced technologies. In the academic realm, this perspective is increasingly supported by empirical evidence. The students’ attitudes towards translation technology and their motivation to learn as well as their intellectual engagement in learning activities were significantly impacted, as demonstrated by Li et al. (2024). Zhang and Li (2024) observed that students’ actions in gamified environments were strongly driven by AI tools’ perceived functional value. Xu et al. (2024) validated the hypothesis that strong performance beliefs associated with smart classrooms directly influence motivational strength and engagement behaviors. Nie et al. (2024) highlighted that belief about one’s performance serves as a cornerstone in the formation of motivational and self-regulatory learning characteristics.

*H3*: PE positively influences LM

#### 2.2.3 Feedback overload (FO)

Feedback Overload (FO) arises when students encounter excessive, redundant, or contradictory inputs from AI-driven or digital learning systems. This phenomenon strains learners’ cognitive bandwidth and may result in psychological overexertion. According to Cognitive Load Theory (Paas et al., 2003; Sweller, 1988), learners have a limited capacity for processing feedback, and when that capacity is overwhelmed, it causes cognitive overload and fatigue. Paterson et al. (2020) found that unfiltered feedback, often seen in AI-based systems, can produce learner disengagement and stress. Similarly, Lipnevich and Panadero (2021) emphasized that feedback must align with learner needs and capacity, or else it becomes a burden rather than a facilitator. Importantly, Feedback Overload is not only fatiguing, it also deteriorates motivational pathways. Alavi (2024) argued that excessive feedback interferes with autonomy and competence, two drivers of intrinsic motivation in self-determined learning. Li and Han (2024) further supported this by showing how poorly timed or dense feedback disrupts learners’ ability to focus, reducing their engagement and intrinsic drive. Additionally, Adanyin (2024) observed that AI-generated feedback loops, when unmanaged, can erode psychological readiness and deter learner perseverance. Huang et al. (2023) also documented how feedback saturation diminishes learning satisfaction, further confirming this motivational erosion. Accordingly, we propose that

*H4*: FO positively influences AF

*H5*: FO negatively influences LM

#### 2.2.4 AI learning anxiety (LA)

Artificial Intelligence Learning Anxiety (LA) describes anxiety arising from a learner’s apprehension regarding the use of AI technologies in an educational context. It results from the learner’s lack of knowledge of the AI tools, failure apprehension, or the burden of the technology itself. If prolonged, anxiety of this sort is capable of inducing cognitive overload, emotional burnout, and ultimately psychological fatigue. The link between anxiety in the academic context and fatigue is chronic and well-documented. In the context of AI-related anxiety, Wang and Wang (2022) developed and validated a specialized scale. They confirmed the scale’s validity and reported that AI anxiety significantly negatively impacts learner engagement and participation. Kim et al. (2023) linked AI anxiety to lower levels of well-being and higher levels of emotional burnout in digital learning environments. In the healthcare sector, Hilty et al. (2022) demonstrated that technology-related anxiety contributes directly to cognitive fatigue. Fernandes and Oliveira (2024) suggested that when anxiety is added to digital overload, fatigue-like symptoms appear due to anger disengagement response. Alavi (2024) noted that anxiety interrupts self-determined processes and drains psychological energy. Based on these arguments, this study proposes that:

*H6*: LA positively influences AF

#### 2.2.5 Learning motivation (LM)

Learning engagement behavior (LEB) is, in practical terms, driven by the psychological concept of learning motivation. Motivation in the intrinsic and extrinsic form constitutes the psychological force that activates, directs, and sustains learner behavior within the classroom. Motivated students exert greater effort, persistence, and involvement, which translate into stronger engagement behaviors characterized by attention, participation, and sustained task involvement. This connection is embedded in self-determination theory which argues motivation improves a learner’s capacity for autonomy and involvement (Deci and Ryan, 2012). Recent studies in technology-enhanced learning further suggest that motivational engagement in digital environments depends on learners’ ability to regulate both cognition and emotion while interacting with adaptive systems (Stalmach et al., 2025). Such findings reinforce the relevance of self-determination theory in understanding psychologically sustainable engagement within AI-supported educational contexts. Empirically, Schaufeli et al. (2002) conceptualizes engagement as vigor, dedication, and absorption, a state variable that is heavily conditioned by motivational readiness. Zhang and Li (2024) corroborate this, demonstrating that students with a higher motivational drive tend to show greater behavioral and cognitive engagement with online learning, particularly with interactive and gamified components. Self-motivation has been shown to act as a mediator between perceived engagement and academic performance in online project-based learning environments (Afzal and Crawford, 2022). In the same vein, Xu and Feng (2024) noted that motivational profiles characterized by resilience and growth mindset tend to forecast positively engagement and scholarly achievement. Nie et al. (2024) highlight that motivated learners display greater emotional and behavioral engagement. To build the rationale on this, we postulate that:

*H7*: LM positively influences LEB

Learning motivation (LM) provides a powerful mediating pathway between both psychological enablers (TSE, PE) and active learning engagement behavior (LEB). While TSE reflects a learner’s belief in their ability to use technology effectively, it is motivation that transforms this belief into effortful engagement. Bandura (1997) emphasized that self-efficacy shapes motivational readiness, which precedes sustained behavior. Compeau and Higgins (1995) noted that even highly confident users relied on motivation to carry intention into practice. Studies by Wang et al. (2022), Xu et al. (2024), and Nie et al. (2024) demonstrate that TSE enhances self-regulated learning, but only when mediated by internalized motivation. Chang and Sun (2024) found AI-related efficacy enhances goal orientation and effort, yet this effort arises through motivational intent. In the case of PE, the belief in AI’s usefulness triggers engagement when filtered through motivation. According to Venkatesh and Davis (2003), PE increases behavioral intention, but motivation channels this intention into engagement. Empirical evidence from Li et al. (2024) and Zhang and Li (2024) confirm that performance beliefs foster emotional and cognitive engagement via motivational routes. Motivation acts as the proximal mechanism through which expectancy beliefs shape persistence and focus (Cai and Yu, 2024; Rahimi, 2024).

Conversely, FO functions as a motivational inhibitor. Although FO initially induces cognitive fatigue (Alavi, 2024; Huang et al., 2023), its indirect route to disengagement operates by eroding motivation. When feedback becomes overwhelming, learners’ autonomy and perceived competence are compromised (Li and Han, 2024), weakening intrinsic motivation. As noted by Adanyin (2024), saturated feedback impairs motivational clarity and reduces intention to act. Therefore, FO does not merely fatigue learners, it disrupts the very mechanism that would drive them to stay engaged. This positions motivation as the mediating force not only for enablers but also for inhibitors, transforming the engagement pathway into a dynamic psychological process. By keeping all such arguments in a pattern, this research hypothesizes that:

*H8*: LM mediates the relationship between TSE and LEB

*H9*: LM mediates the relationship between PE and LEB

*H10*: LM mediates the relationship between FO and LEB

#### 2.2.6 AI fatigue (AF) as a symptomatic strain

AI fatigue can be related to the emotional and cognitive exhaustion experienced by users due to sustained interaction with AI-powered or digital technologies. It arises when the mental demand of navigating AI tools exceeds the users’ psychological and attentional resources, resulting in motivational decline and behavioral withdrawal. Learning engagement behavior can be significantly deteriorated by fatigue in digital and AI learning environments. It is defined as an ongoing mental effort to engage in learning activities, where the learner’s focus is impaired due to emotional depletion and self-activation. The erosion of cognitive and emotional resources as a result of fatigue affects attention, motivation, and persistence, which are foundational elements of engagement. As Schaufeli et al. (2002) explained, engagement is a state of vigor, dedication, and absorption, conditions incompatible with fatigue. Huang et al. (2023) demonstrated mobile app fatigue diminishes willingness to engage with educational materials. Fernandes and Oliveira (2024) linked social media fatigue with reduced participation. Hilty et al. (2022) emphasized that AI-induced fatigue correlates with reduced emotional engagement. Robert Selvam et al. (2023) and Song et al. (2023) confirmed that platform-related exhaustion suppresses learning behavior. There we propose that

*H11*: AF negatively influences LEB

AI learning anxiety (LA) erodes learners’ psychological readiness, reducing participation in digital educational settings. This relationship often unfolds through emotional and cognitive fatigue, which diminishes learning engagement behavior (LEB). Fatigue functions as a mediating mechanism: anxiety undermines cognitive capacity, leading to disengagement. Cognitive-affective stress models support this, linking anxiety to emotional exhaustion and impaired learning. Wang and Wang (2022) observed that learners with AI-specific anxiety reported psychological strain and reduced participation. Hilty et al. (2022) confirmed that interface-related anxiety contributes to AI fatigue. Kim et al. (2023) emphasized that AI anxiety disrupts motivational continuity via emotional exhaustion. Fernandes and Oliveira (2024) added that technology-induced anxiety precipitates fatigue, which then leads directly to behavioral withdrawal. Huang et al. (2023) demonstrated that stress-induced fatigue mediates reduced digital tool usage. Chang and Sun (2024) and Alavi (2024) confirmed anxiety-driven fatigue interrupts self-regulation and erodes sustained effort. Thus, fatigue transforms anxiety into disengagement.

Feedback overload, too, leads to disengagement, primarily through fatigue. AI environments deliver real-time, complex feedback that burdens cognitive resources. Lipnevich and Panadero (2021) warned that oversaturation of feedback induces anxiety and mental strain. Paterson et al. (2020) found that inconsistent feedback reduces learner motivation. Lee and Kim (2021) demonstrated that excessive, especially negative, feedback causes emotional exhaustion and disengagement. Song et al. (2023) noted mental fatigue and demotivation from saturated feedback. Li and Han (2024) observed that learners disengage not due to apathy, but from fatigue induced by automated feedback cycles. Huang et al. (2023) linked feedback-induced fatigue to dropout behavior. Szumowska et al. (2023) confirmed that feedback processing drains psychological energy, weakening learning effort. Thus, fatigue bridges feedback saturation and disengagement by depleting learners’ emotional and cognitive reserves. Therefore, we hypothesize that:

*H12*: AF mediates negative relationship between LA and LEB

*H13*: AF mediates negative relationship between FO and LEB

**Figure 1:**
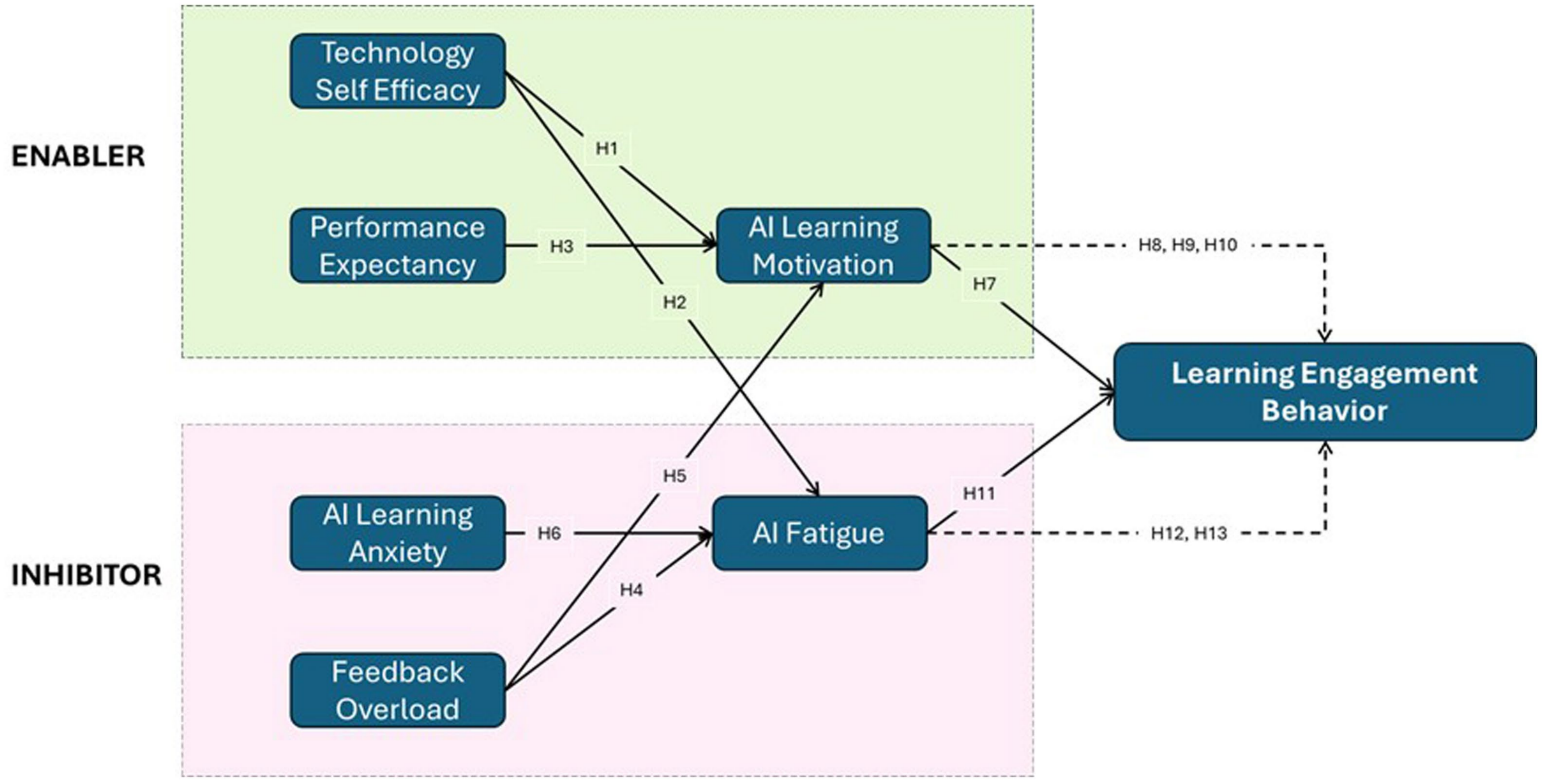
Research framework.

Figure 1 presents the research framework, which models how enablers (technology self-efficacy and performance expectancy) and inhibitors (AI learning anxiety and feedback overload) influence learning engagement behavior. Learning motivation and AI fatigue serve as mediators that explain the psychological pathways linking these factors to engagement outcomes.

## 3 Methodology

This study adopts a quantitative, cross-sectional survey design to examine the enablers and inhibitors of learning engagement behavior (LEB) in AI-enhanced educational environments. The research focuses on how technology self-efficacy (TSE), performance expectancy (PE), learning motivation (LM), AI learning anxiety (LA), feedback overload (FO), and AI fatigue (AF) interact to influence student engagement. A structured self-administered questionnaire was employed to measure these constructs and test the theoretical model using Partial Least Squares Structural Equation Modeling (PLS-SEM).

The study targeted active learners who possessed verified, hands-on experience utilizing AI-powered educational tools within the 6 months preceding data collection. To ensure sample relevance and methodological rigor, explicit purposive screening criteria were enforced via the online survey instrument. Respondents were required to pass a two-stage filter: (1) confirming regular, interactive use of at least one adaptive platform class, such as generative AI assistants, automated language learning apps, or intelligent tutoring systems, and (2) demonstrating sufficient language proficiency to fully comprehend the survey items. Out of 351 total responses received via online educational communities and distribution channels, exactly 100 cases were excluded because they failed to meet these strict inclusion rules. This rigorous screening process resulted in a final dataset of 251 valid respondents suitable for robust structural equation modeling. Data were collected from 251 valid respondents, exceeding the threshold for complex structural models involving multiple latent variables (Hair et al., 2014).

The questionnaire drew from validated instruments: TSE from Compeau and Higgins (1995), PE from Venkatesh and Davis (2003), LM from Deci and Ryan (2012), LA from Wang et al. (2024), FO from Fernandes and Oliveira (2024), AF from Kim et al. (2023), and LEB from Schaufeli et al. (2002). All items were measured on a 5-point Likert scale (1 = Strongly Disagree to 5 = Strongly Agree). Items were adapted slightly to fit the context of AI-supported learning without altering construct meaning. To establish face validity, the questionnaire was reviewed by five domain experts in educational technology and behavioral learning sciences. Based on their feedback, modifications were made to improve clarity, relevance, and item specificity. A pilot test with 30 participants was then conducted to assess reliability and measurement adequacy. Cronbach’s alpha, composite reliability, and average variance extracted (AVE) all exceeded recommended cutoffs, supporting the instrument’s psychometric properties (Fornell and Larcker, 1981).

Data were collected through an online survey administered via Google Forms. A professional data collection agency managed recruitment to ensure relevance and quality. Participants were required to be active users of AI tools and have sufficient English proficiency to understand the survey. Participation was voluntary, with informed consent obtained from all respondents. Anonymity and confidentiality were strictly maintained.

Ethical guidelines related to data use, participant rights, and confidentiality were followed throughout (Resnik, 2024). Data analysis was performed using SmartPLS v4. The measurement model was assessed for reliability and validity using Cronbach’s alpha, CR, and AVE. Discriminant validity was confirmed using Fornell–Larcker criteria and HTMT ratios. The structural model was evaluated through path coefficients, and *R*^2^ values (Hair et al., 2019; Sarstedt et al., 2022). Mediation effects were tested via indirect path analysis, allowing examination of both direct and crossover mechanisms.

The potential presence of common method bias in the collected data was assessed by Harman’s single-factor test. The results revealed that the first factor accounted for 35.86% of the total variance. Its value is well below the recommended threshold of 50%. This signals that common method bias is unlikely to pose a serious threat to the validity of the findings. No demographic control variables were included in the structural model because the primary objective of the study was to examine the theorized psychological relationships among the focal constructs within the proposed crossover framework.

## 4 Results

### 4.1 Demographic profile of respondents

The gender distribution was relatively balanced, with 120 females and 131 males. The majority of respondents (84.3%) were aged between 18 and 34, indicating a predominantly young adult sample. Most participants held at least a bachelor’s degree (62.1%), and a significant portion specialized in Engineering/Technology (62.8%), followed by Business/Management (24.1%). In terms of learning environments, the respondents were engaged across various contexts: 35.3% in self-paced informal learning, 40.2% in formal university/college settings, and 24.5% in professional training programs. Experience with AI learning tools was notably high, with 59.4% using them frequently and 28.7% occasionally. Similarly, self-directed learning habits were prevalent, with over 75% reporting frequent or very frequent engagement. Digital usability confidence was strong among respondents, with 140 identifying as “very comfortable” and another 89 as “comfortable.” Regarding preferred AI tools, language learning apps (48.3%) and skill development platforms (33.7%) were the most commonly used, while fewer respondents reported using AI-powered tutoring systems or adaptive learning platforms. Table 1 shows the detailed breakdown of the demographics.

**Table 1:** Demographics results.

Gender		AI learning experience	
Female	120	Infrequently	21
Male	131	Yes, frequently	155
Age		Yes, occasionally	75
18–24	116	Self-directed learning experience	
25–34	101		
35–44	23	Never	2
45 = 54	4	Often	81
55 and above	3	Rarely	6
Under 18	4	Sometimes	47
Education		Very often	115
Some college courses/Diploma	22	Digital usability comfort	
Bachelor’s degree	160	Comfortable	89
Master’s degree	51	Neutral	21
Doctorate degree	10	Uncomfortable	1
Other	8	Very Comfortable	140
Area of specialization		AI learning tools	
Engineering/Technology	164	Language learning apps	124
Business/Management	61	Skill development platforms	87
Social Sciences	7	AI-powered tutoring systems	14
Arts/Humanities	8	Adaptive learning platforms	9
Natural Sciences	8	Other	17
Other	3		
Learning environment			
Personal/Informal Learning (Self-paced)	88		
Professional Training/Development	61		
University/College (Formal Education)	102		

### 4.2 PLS-SEM results

#### 4.2.1 Measurement model

The measurement model was evaluated for internal consistency reliability, convergent validity, and construct reliability, as recommended by Hair et al. (2019). As per Table 2, all constructs demonstrated strong internal consistency, with Cronbach’s Alpha values ranging from 0.817 (FO) to 0.957 (PE), exceeding the minimum threshold of 0.70. Composite reliability (CR) values were also robust, falling between 0.879 and 0.964, surpassing the recommended benchmark of 0.70, indicating high construct reliability. Convergent validity was confirmed as all Average Variance Extracted (AVE) values were above the acceptable threshold of 0.50, ranging from 0.644 (FO) to 0.794 (PE). These results support the unidimensionality and validity of the measurement scales used. Discriminant validity was verified using the HTMT (Heterotrait-Monotrait) ratio of correlations. All HTMT values were below the conservative threshold of 0.85, with the highest observed value being 0.84 between TSE and PE, which still supports acceptable discriminant validity. Notably lower HTMT values (e.g., 0.095 between AF and LEB, 0.086 between LA and LM) further affirm distinctiveness among constructs.

**Table 2:** Reliability and validity results.

Variable	Alpha	C.R	AVE	HTMT Ratio of Correlation						
				AF	FO	LA	LEB	LM	PE	TSE
AF	0.87	0.91	0.72							
FO	0.81	0.87	0.64	0.65						
LA	0.92	0.94	0.66	0.67	0.53					
LEB	0.88	0.91	0.64	0.09	0.39	0.23				
LM	0.93	0.94	0.75	0.10	0.40	0.08	0.73			
PE	0.95	0.96	0.79	0.15	0.35	0.11	0.56	0.79		
TSE	0.93	0.94	0.78	0.14	0.40	0.08	0.44	0.70	0.84	

As per Table 3, all indicator outer loadings exceeded the recommended threshold of 0.70, confirming indicator reliability (Hair et al., 2019). Loadings ranged from 0.730 (LA1) to 0.912 (PE3), indicating that each item strongly represents its respective construct. No indicators required removal, as all contributed meaningfully to construct measurement. Overall, the model demonstrates strong measurement properties, indicating that all constructs are reliable, valid, and distinct from one another, making them suitable for structural model analysis (Hair et al., 2021).

**Table 3:** Outer loadings results.

Variables	Items	Loadings	Variables	Items	Loadings
AI Fatigue (AF)	AF1	0.818	Learning motivation (LM)	LM1	0.838
	AF2	0.894		LM2	0.859
	AF3	0.869		LM3	0.901
	AF4	0.826		LM4	0.853
Feedback overload (FO)	FO1	0.778		LM5	0.878
	FO2	0.806		LM6	0.868
	FO3	0.792	Performance expectancy (PE)	PE1	0.882
	FO4	0.833		PE2	0.894
AI learning anxiety (LA)	LA1	0.73		PE3	0.912
	LA2	0.819		PE4	0.907
	LA3	0.831		PE5	0.871
	LA4	0.856		PE6	0.867
	LA5	0.833		PE7	0.904
	LA6	0.857	Technology self-efficacy (TSE)	TSE1	0.86
	LA7	0.822		TSE2	0.906
	LA8	0.763		TSE3	0.907
Learning engagement behavior (LEB)	LEB1	0.799		TSE4	0.874
	LEB2	0.82		TSE5	0.877
	LEB3	0.833			
	LEB4	0.744			
	LEB5	0.744			
	LEB6	0.869			

#### 4.2.2 Structural model

**Figure 2:**
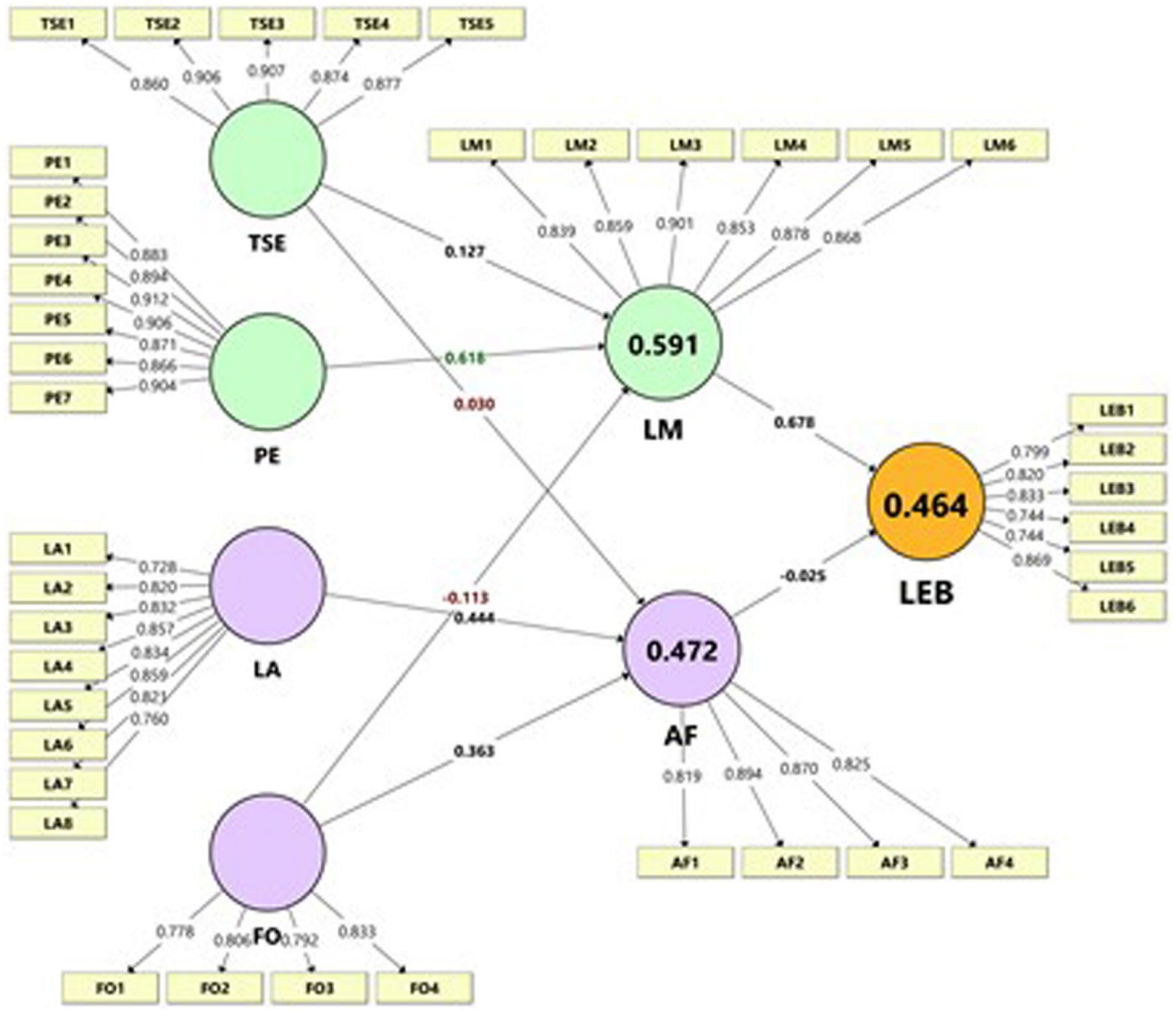
PLS-SEM framework.

The structural model was evaluated using *R*^2^ values to determine the model’s explanatory power for each endogenous construct. As illustrated in Figure 2, the model accounted for 59.1% of the variance in Learning Motivation (LM), 47.2% in AI Fatigue (AF), and 46.4% in Learning Engagement Behavior (LEB). According to Hair et al. (2019), these *R*^2^ values indicate moderate to substantial explanatory power, particularly for LM, which emerged as the most strongly explained construct in the model. These results demonstrate that the proposed predictors are effective in explaining the variance in the core constructs of the study.

All endogenous constructs exhibit predictive relevance, as indicated by *Q*^2^ values greater than zero (see Table 4). LM records the strongest predictive relevance (0.431), followed by AF (0.332) and LEB (0.29), reflecting substantial explanatory power for these constructs. Effect size results show that LM exerts a large influence on LEB (*f*^2^ = 0.851), while FO and LA have moderate effects on LM (*f*^2^ = 0.168 and 0.286, respectively) as shown in Table 4. Other relationships demonstrate small or trivial effects. That indicates minimal contribution to the variance explained. These patterns highlight LM’s central role in driving LEB.

**Table 4:** *f*-squares and *Q*-squares results.

Constructs	*Q* ^2^	*f*-square		
		AF	LM	LEB
AF	0.332			0.001
FO		0.168	0.027	
LA		0.286		
LEB	0.29			
LM	0.431			0.851
PE			0.344	
TSE		0.001	0.014	

As described in Table 5, the strongest significant relationship was from Learning Motivation (LM) to Learning Engagement Behavior (LEB) (H7, *β* = 0.679, *t* = 13.783), indicating LM as a key predictor of engagement. This was followed by Performance Expectancy (PE) to LM (H3, *β* = 0.619), and LA to AF (H6, *β* = 0.444), both showing substantial direct effects. Feedback Overload (FO) had significant paths both toward AF (H4, *β* = 0.363) and negatively toward LM (H5, *β* = −0.113). The indirect effect of PE → LM → LEB (H9) was also significant (*β* = 0.42), highlighting a meaningful mediating role of LM. Additionally, H10 revealed a significant negative indirect effect from FO to LEB via LM (*β* = −0.077), suggesting that increased feedback overload may reduce engagement through its impact on motivation. In contrast, the weakest and non-significant paths included TSE → AF (H2, *β* = 0.03), AF → LEB (H11, *β* = −0.025), and several not supported mediation paths (H8, H12–H13), all with low coefficients and insignificant *t*-values.

**Table 5:** Hypotheses results.

Hypothesis	Path	Beta	*T* stat	*p*-values	Decision
H1	TSE → LM	0.127	1.115	0.133	Reject
H2	TSE → AF	0.03	0.552	0.291	Reject
H3	PE → LM	0.619	4.868	0.000	Accepted
H4	FO → AF	0.363	5.212	0.000	Accepted
H5	FO → LM	−0.113	2.188	0.015	Accepted
H6	LA → AF	0.444	6.667	0.000	Accepted
H7	LM → LEB	0.679	13.783	0.000	Accepted
H8	TSE → LM → LEB	0.086	1.113	0.133	Reject
H9	PE → LM → LEB	0.42	4.655	0.000	Accepted
H10	FO → LM → LEB	−0.077	2.078	0.019	Accepted
H11	AF → LEB	−0.025	0.461	0.323	Reject
H12	LA → AF → LEB	−0.011	0.448	0.327	Reject
H13	FO → AF → LEB	−0.009	0.45	0.326	Reject

## 5 Discussion

This study examined how psychological enablers and inhibitors jointly shape learning engagement behavior in AI-mediated environments. Drawing on Self-Determination Theory (Deci and Ryan, 2012), Social Cognitive Theory (Bandura, 1986), and Cognitive Load Theory (Sweller, 1988), we developed a crossover model in which technology self-efficacy interacts with stressors such as AI fatigue. Learning motivation and AI fatigue were positioned as key mediators, representing motivational activation and cognitive depletion, respectively. Out of 13 hypotheses tested, 7 were supported and 6 were not supported based on statistical significance (*p* < 0.05). The model explained 59% of the variance in learning motivation, 47% in fatigue, and 46% in engagement behavior, offering strong support for the psychological interdependence between enabling beliefs and inhibitory strain.

The study began by testing how Technology Self-Efficacy (TSE) influences learning outcomes. Surprisingly, TSE had no significant effect on either motivation (H1) or AI fatigue (H2). This finding is less consistent with much of the prior social cognitive literature linking efficacy beliefs to learning behavior (Deci and Ryan, 2012), (Compeau and Higgins, 1995). The result suggests that simply feeling confident with technology may not be enough unless it is accompanied by a clear belief in the system’s value (Chang and Sun, 2024; Liwanag and Galicia, 2023). In contrast, Performance Expectancy (PE) showed strong effects on both motivation (H3) and indirectly on engagement. Learners who believed AI tools would improve academic outcomes became significantly more motivated (Xu et al., 2024; Zhang and Li, 2024). This affirms UTAUT’s core proposition that perceived usefulness is key to adoption (Venkatesh and Davis, 2003). Among inhibitors, Feedback Overload (FO) had two direct effects: it increased fatigue (H4) and reduced motivation (H5). These results confirm that overwhelming feedback does more than tire students, it undermines their psychological readiness to engage (Alavi, 2024; Huang et al., 2023). AI Learning Anxiety (LA) also significantly raised fatigue (H6), aligning with research that links technology-related anxiety to emotional depletion (Kim et al., 2023; Wang and Wang, 2022).

Learning Motivation (LM) proved to be the most potent driver of engagement behavior (H7). When motivation was high, learners sustained effort, focus, and emotional involvement, confirming self-determination theory’s claim that purpose and autonomy are vital for sustained engagement (Deci and Ryan, 2012; Schaufeli et al., 2002). Similarly, when motivation is negatively influenced by overarching feedback, the engagement lessens. This reflects the negative role of feedback on engagement through decreased motivation (H10). Interestingly, AI Fatigue (AF) did not exhibit a statistically significant direct effect on learning engagement behavior in the present model. This finding should be interpreted cautiously rather than as evidence that fatigue is irrelevant in AI-supported learning contexts. Fatigue remained significantly associated with both feedback overload and AI learning anxiety, indicating its continued psychological importance within the broader learning experience. One possible explanation is that fatigue alone may not immediately translate into observable disengagement behavior, particularly among learners who remain academically motivated despite experiencing cognitive strain. Another possibility is that the effects of fatigue may emerge more strongly over longer periods of sustained AI interaction than could be captured through the present cross-sectional design. Accordingly, the findings suggest that fatigue may function more as an early indicator of psychological strain than as an immediate behavioral determinant of disengagement.

The mediation tests brought key psychological clarity. Motivation significantly mediated the effect of performance expectancy on engagement (H9), demonstrating that belief in performance must first energize motivation before behavior follows (Li et al., 2024). However, the pathway from TSE to engagement via motivation (H8) was not supported, aligning with the direct effects.

### 5.1 Bounding factors and the role of technology self-efficacy

The structural model yielded non-significant paths for H1, H2, and the corresponding mediation pathway H8. While prior educational technology literature frequently associates operational confidence with a higher motivational drive, these findings suggest that within advanced AI-mediated learning environments, the influence of technology self-efficacy is highly context-dependent rather than universally stable. This variance can be explained by two main factors. First, our demographic profile reveals a sample with exceptionally high baseline digital familiarity: 91.2% of respondents identified as “comfortable” or “very comfortable” with digital usability, and 62.8% originated from Engineering and Technology specializations. For a highly technically oriented cohort, navigating the user interfaces of modern AI systems represents a normalized baseline characteristic rather than an active motivational differentiator.

Second, as intelligent educational tools become more autonomous and adaptive, the learner’s inner psychological world shifts focus from operational execution to instructional outcome. Operational confidence (TSE) remains a latent prerequisite, whereas situational value—captured by Performance Expectancy (PE, *β* = 0.619, *p* < 0.001) becomes the primary active driver that triggers internal motivational states. Consequently, technical self-efficacy may appear inert in cross-sectional models unless it is explicitly paired with high perceived educational utility. Furthermore, neither anxiety nor feedback overload translated into disengagement through fatigue (H12, H13), suggesting that fatigue alone may not directly translate into observable disengagement behavior within the present learning context. The most insightful result came from H13, where motivation mediated the negative effect of feedback overload on engagement. This confirms that inhibitors erode behavior not by draining energy alone, but by weakening the psychological fuel that drives effort (Adanyin, 2024; Szumowska et al., 2023). Overall, the model reveals that engagement emerges from the interplay of enabling beliefs, disruptive stressors, and the fragile dynamics of motivation. However, the cross-sectional nature of the study requires caution in interpreting directional or causal relationships among the constructs.

### 5.2 Theoretical contribution

This study advances theory on learner engagement in AI-enhanced educational environments by reframing it through a crossover enabler–inhibitor lens. Prior work has typically treated enablers and inhibitors as discrete or parallel forces (Cenfetelli, 2004; Liang et al., 2025), offering only partial accounts of how learners interact with intelligent systems. We move beyond this bifurcation by showing that enablers and inhibitors co-construct engagement outcomes through a shared psychological space.

Our model demonstrates that motivation, rather than being a mere outcome, is both a product and casualty of the tension between enabling beliefs and cognitive-emotional strain. This theoretical repositioning contributes to a more nuanced understanding of engagement as a dynamic interplay between motivational ignition and depletion.

We refine existing technology acceptance models by illustrating that performance expectancy (PE) requires motivational mediation to translate into behavioral engagement. While the UTAUT model (Venkatesh and Davis, 2003) has long emphasized PE as a key predictor of technology use, our findings show that its influence on actual engagement is not direct, but rather channeled through learning motivation. This extends prior work by Zhang and Li (2024) and Xu et al. (2024), who noted strong expectancy-engagement links but did not isolate the motivational mechanism. Our results suggest that the perception of performance value must energize internal motivational states before behavioral change can occur, advancing both UTAUT and expectancy-value frameworks into the affective-cognitive domain.

The model disrupts conventional assumptions from social cognitive theory. Technology self-efficacy (TSE), often regarded as a robust predictor of self-regulated learning (Deci and Ryan, 2012), Schunk and DiBenedetto (2020), showed no significant impact on motivation or fatigue in our analysis. These findings suggest that the relationship between efficacy beliefs and engagement may be more context-sensitive than traditionally assumed, particularly within AI-mediated learning environments characterized by high baseline digital familiarity and autonomy.

We offer new theoretical ground for understanding the disruptive role of feedback overload (FO). Existing cognitive load theory posits that extraneous information reduces working memory capacity and learning efficiency (Paas et al., 2003; Sweller, 1988). We extend this model by showing that FO not only induces fatigue, but also depletes motivation. The crossover effect is key: FO erodes engagement not simply by exhausting cognitive resources, but by attacking the psychological fuel that sustains behavioral effort (Alavi, 2024), (Huang et al., 2023). This positions FO as a dual-channel inhibitor, affecting both the body (fatigue) and the will (motivation), thereby demanding more sophisticated feedback design in AI learning systems.

The study clarifies the limited behavioral power of fatigue. Despite its theoretical prominence in disengagement models, fatigue did not significantly reduce engagement when examined in isolation. This challenges assumptions in affective computing and cognitive strain models, suggesting that fatigue becomes behaviorally relevant only when it co-occurs with motivational erosion (Fernandes and Oliveira, 2024; Szumowska et al., 2023). This finding elevates motivation as the primary determinant of learning engagement and reframes fatigue as a secondary, symptomatic construct. It also reinforces self-determination theory’s argument that agency and purpose, not just stamina, drive sustained effort (Deci and Ryan, 2012).

The paper contributes to enabler–inhibitor theory by developing a psychologically integrated crossover model. Previous studies [e.g., (Balakrishnan et al., 2024; Liu et al., 2025)] identified separate antecedents of adoption or discontinuance but did not theorize their interaction. Our model demonstrates that inhibitors such as FO and AI anxiety can actively suppress the effect of enablers like PE by intercepting the motivational pathway. This moves the literature beyond dual-path frameworks to a more ecologically valid understanding of how learners actually experience AI systems, through overlapping psychological forces that compete for control over behavior.

### 5.3 Practical contribution

This study offers significant insights for the design and deployment of AI-enabled learning environments. While much of the existing literature emphasizes technological affordances and system usability, our findings emphasize the importance of psychological alignment. Engagement in AI-mediated contexts cannot be secured through intelligent automation alone. It depends on how learners internalize, interpret, and respond to cognitive and emotional cues embedded in the system. In practice, this calls for a more nuanced, learner-centered approach to AI integration, one that aligns technological functionality with motivational design. The central finding, that learning motivation is the strongest predictor of behavioral engagement, reinforces the need to prioritize motivational architecture in AI learning systems. This goes beyond content relevance or usability. It points to the learner’s experience of purpose, autonomy, and progress as fundamental conditions for sustained engagement. Systems should be built to enhance not just task efficiency, but also psychological investment. Learning tools must signal value, support goal-setting, and provide timely affirmations of progress. Designers must embed motivational scaffolds that help learners experience competence, not just task completion.

This study also redefines the role of feedback. While traditionally regarded as a supportive feature, feedback becomes disruptive when excessive or poorly structured. The finding that feedback overload not only contributes to fatigue but also undermines motivation indicates a need for recalibration. Effective systems should regulate feedback in both quantity and timing. Rather than continuous output, feedback should be context-sensitive, learner-paced, and cognitively digestible. Such moderation preserves clarity and reduces informational strain. Developers should consider feedback compression algorithms or learner-controlled feedback toggles as core components of AI interaction design. Contrary to widespread assumptions, technology self-efficacy did not significantly influence motivation or fatigue. This challenges conventional training models that emphasize digital confidence as a primary goal. Our results suggest that confidence without perceived value may lack behavioral power. Learners must not only feel capable but also perceive the system as meaningful. This distinction has strong implications for onboarding and instructional design. Training should combine skill-building with explicit value framing, helping learners understand not just how to use the system, but why it supports their goals. Without this alignment, efficacy may not translate into engagement.

The study also highlights the importance of monitoring psychological strain within AI-supported learning systems. Although AI fatigue did not directly predict disengagement behavior in the structural model, it remained strongly associated with anxiety and feedback overload. This suggests that fatigue may represent an early psychological warning signal rather than an immediately observable behavioral outcome. From a practical perspective, monitoring fatigue-related indicators may help institutions identify emerging emotional strain before it develops into more serious disengagement, reduced learning quality, or long-term burnout. Subtle behavioral patterns, such as erratic pacing, repeated retries, or abrupt pauses in activity, may therefore provide valuable signals for supportive intervention within adaptive learning systems.

At the system level, these findings suggest a broader shift in how educational technologies should be evaluated. Performance metrics alone are insufficient. Institutions should assess systems based on their psychological climate, the extent to which they preserve energy, support motivation, and buffer cognitive strain. Learner experience audits, incorporating emotional usability and motivational profiling, can provide more meaningful insights than traditional usage logs. Technology that optimizes efficiency but fails to support internal readiness may ultimately contribute to disengagement or burnout.

Furthermore, the results carry implications for institutional support structures. Most student support models are reactive. They wait for failure to trigger intervention. Yet fatigue and motivational decline often unfold silently. Institutions should consider proactive support models that anticipate strain. These may include embedded coaching features, just-in-time feedback moderation, or automated nudges that restore focus and motivation. Support must be integrated, continuous, and unobtrusive. The goal is to maintain learner vitality, not just to correct performance lapses. Lastly, the findings challenge the assumption that engagement is a stable outcome. Engagement emerges from dynamic tensions between enablers and inhibitors. It is not a matter of system adoption alone, but of sustained psychological calibration. Designing for engagement thus requires interdisciplinary thinking. Educational technologists, learning scientists, and cognitive psychologists must collaborate to create environments that are not only smart but also psychologically sustainable. This includes building systems that protect attention, reinforce purpose, and adapt not only to performance data but to patterns of motivational strain.

These findings also inform concrete design directions for AI-enabled learning environments. Such as motivation-aware dashboards could frame task value, set incremental goals, and surface timely progress cues, ensuring that engagement is anchored in perceived purpose rather than technical confidence alone. Moreover, adaptive feedback regulators could dynamically adjust the quantity and timing of system feedback based on real-time indicators of cognitive load, thereby preventing overload and protecting motivational resources. Furthermore, embedded anxiety-monitoring features could identify subtle signs of emotional strain and activate light-touch recovery mechanisms, such as delayed feedback, quiet modes, or guided micro-breaks. Collectively, these design approaches align system functionality with the psychological conditions necessary for sustained learner engagement.

### 5.4 Limitation and recommendation

This study offers a grounded framework for understanding psychological mechanisms driving engagement in AI-enhanced learning. However, the use of a cross-sectional design constrains the ability to examine causal relationships or temporal shifts in motivation and fatigue. Although the structural model reveals strong predictive associations, the dynamic nature of engagement likely unfolds over time. Longitudinal or real-time tracking methods would provide deeper insight into how enabler and inhibitor effects accumulate or change across learning phases. The sample was drawn from learners already familiar with AI-based tools, which may have influenced how they interpreted self-efficacy, overload, or anxiety. Learners with limited digital exposure or different educational backgrounds may experience these constructs differently. Future work should incorporate more diverse populations to explore how digital readiness or prior experience conditions the psychological response to AI systems.

In addition, the sample was heavily represented by learners from Engineering and Technology backgrounds. Such participants may possess greater familiarity and comfort with AI systems than learners from other disciplinary areas. This may have influenced the observed relationships, particularly those involving technology self-efficacy. Future research should examine whether the proposed framework operates similarly across humanities, social sciences, and less technology-intensive educational settings. All psychological constructs were measured through self-report, introducing the possibility of subjective bias or inflated associations. While validated scales were used, self-perception may not fully align with actual behavior or affective states. Future studies would benefit from integrating behavioral data from learning systems or physiological indicators of fatigue and stress to validate psychological self-assessments. The model emphasized individual cognitive and emotional factors but did not include broader ecological elements such as instructor behavior, peer support, or platform usability variations. These factors may interact with personal beliefs and emotional states to shape engagement trajectories. Incorporating social, institutional, and design-level variables would provide a more complete understanding of engagement within complex learning systems.

Future research should also examine how AI tools can respond adaptively to motivational decline or early signs of cognitive strain. Building intelligent systems that detect emotional shifts and personalize pacing, feedback, or challenge levels holds promise for advancing both theory and practice in digitally mediated learning. Future research should engage with deploy-and-monitor field experiments or within-subject designs to apprehend the temporal dynamics of motivation and fatigue in AI-mediated learning. Integrating learning analytics logs and physiological indicators would deliver objective measures to authenticate self-reported psychological states and reveal patterns not visible through surveys alone.

## 6 Conclusion

This study contributes to a deeper understanding of the psychological mechanisms shaping engagement within AI-supported learning environments. It shows that engagement is an outcome of using the system which is mediated psychologically and is shaped by enabling and constraining factors. Performance expectancy strengthens learning motivation and contributes to sustained engagement behavior. On the contrary, feedback overload decreases motivation, and increases tiredness, disrupting the learner’s cognitive and emotional equilibrium. Motivation to learn emerged as the strongest predictor of engagement behavior, while fatigue was secondary, with minimal direct impact. These results highlight the importance of reconceptualizing engagement in AI contexts as psychological alignment, rather than looking at the interaction count, or task completion metrics. The crossover model proposed here provides a more comprehensive explanation not only for motivation, but also for how systems that disregard cognitive limits or emotional strain disrupt it. For researchers, the findings highlight the importance of studying AI learning environments through multidimensional behavioral models.

Future work should expand on these pathways using longitudinal or real-time data, and include institutional and social factors as contextual moderators. Practitioners, in turn, are provided with the results on the design of AI systems that control feedback, conserve learner energy, and enhance perceived value. Positioning motivation as the central psychological driver contributes both theoretically and practically to the development of intelligent, adaptive, and learner-centered educational systems.

## Data Availability

The raw data supporting the conclusions of this article will be made available by the authors, without undue reservation.
